# Comparison of Axillary versus Rectal Temperature Timing in Canine and Feline Patients

**DOI:** 10.3390/vetsci10070475

**Published:** 2023-07-21

**Authors:** Olivia Beyer, Ashlynn Lueck, Cord Brundage

**Affiliations:** Biology Department, University of Wisconsin La Crosse, 1725 State Street, La Crosse, WI 54601, USA

**Keywords:** temperature, restraint, appointment, procedure duration, axilla, rectal, cat, dog, thermometry

## Abstract

**Simple Summary:**

Numerous studies have tested the accuracy of thermometers in dogs and cats to find alternatives to rectal placement. These studies have not looked at the difference in restraint and pet handling time that may be involved in thermometer selection. Here we tested the time involved in approaching restraining and recording temperatures from 114 dog and 72 cat patients, both under the forelimb (axillary) and rectally, in a standardized clinical environment. We compared animal size, body shape, weight, age, breed, and coat type in both dogs and cats to identify possible contributing factors that may influence these results. The axillary method was significantly shorter than the rectal method in all breeds and groups tested except the Scottish Fold cat breed, which showed no difference. The shorter duration in axillary thermometry was due primarily to reductions in the approach and handling time seen in the axillary trials compared with the rectal trials. In the rectal trials, an increased duration was noted in cats with longer hair than in cats with shorter hair. No other factor tested (i.e., size, age, etc.) played a significant role in the results. This data suggests that one advantage of axillary thermometry over rectal thermometry may be a shorter pet handling and procedure time.

**Abstract:**

Research on alternatives to rectal thermometry in canine and feline patients has focused on equipment and measurement location but not procedure duration. In a crossover clinical scenario, we evaluated the time prior to (Pre-TempT) and after (Post-TempT) rectal and axillary thermometry in a diverse demographic of canine (*n* = 114) and feline (*n* = 72) patients. Equipment duration was controlled to determine a presumptive total time (TTime) associated with each thermometry method. Pre-TempT and TTime were significantly shorter in axillary thermometry trials for both canine and feline pets (*p* < 0.001). There was no difference in Post-TempT between thermometry methods in canine patients (*p* = 0.887); however, the Post-TempT was longer in felines after axillary thermometry *(p* = 0.004). Reductions in Pre-TempT and TTime were not significant in Scottish Fold breed cats. Within the feline rectal trials, the TTime of domestic-long-haired breeds was significantly longer than that of domestic-short-haired breeds (*p* = 0.019). No other tested parameter (i.e., size, body shape, age, weight, breed, coat type, or procedure order) played a significant role in these results. Axillary thermometry was faster than rectal thermometry in both canine and feline pets, primarily due to the time associated with animal approach and restraint (Pre-TempT). These results have implications for optimizing clinic workflow, appointment durations, and patient handling time.

## 1. Introduction

Temperature is one of the vital assessments used to objectively examine a veterinary patient, the results of which can have significant implications for the health status of that patient [1,2,3]. Predictive rectal thermometry is the most common form of thermometry used in veterinary clinical practice, despite 30 years of research on alternative modalities [4,5,6]. Research on alternatives to rectal thermometry has primarily focused on correlating the temperature values of these alternative methods to either rectal temperatures or a core body temperature [4,5,7,8,9]. 

Some of the motivations discussed to identify alternative modalities to rectal thermometry have been the difficulty in obtaining rectal temperatures in uncooperative or fractious animals, the influence of feces or perianal disease processes (i.e., fistulas, tumors, etc.) falsifying results, and the potential increase in the risk of nosocomial infections with shared instrument usage and handling [4,10,11,12]. Rectal thermometry may be more stressful than potential alternatives. Gomart and colleagues (2014) looked at heart rate and graded stress behaviors (vocalization, lip licking, shaking, panting, and defensive behaviors) in 250 dogs following rectal, auricular, and axillary temperature readings. They found that heart rate and stress behavior scores were both significantly increased following rectal thermometry compared with the other methods [13]. In cats, it has been noted that the stress of rectal temperature measurement alone may be enough to elevate the rectal temperature [3].

Stress in the clinical environment can negatively influence not only the current visit but subsequent visits [14,15]. Some inherent stress may be from interactions with practitioners in the clinical setting; this is often referred to as a “white-coat effect” and has been described in both dogs and cats [1,3,16]. Another potential stressor in the clinical environment may be an appointment’s duration. In human literature, one of the main factors adversely affecting patient satisfaction is time spent in a clinic [17,18]. In veterinary medicine, it is widely regarded that minimizing the duration of a procedure helps to minimize stress [19,20]. When feasible, the minimum duration of restraint that can be used for immobilization should be used [14]. Despite the importance of minimizing appointment duration, handling time, and procedure time, an analysis of how the method of thermometry affects the duration of restraint and appointment time for dogs and cats has not been investigated.

Here we compare the time associated with approaching, restraining, and recording the temperature of canine and feline patients with a digital predicative rectal thermometer and a digital axillary thermometer. The instrument duration was kept constant to focus on the temporal aspect of restraining with either method. Innovations and technology continue to advance the speed and quality of instrumentation. Rather than focusing on the type of thermometer, our aim was to compare the temporal aspects of the procedure at each location. 

We hypothesize that there would be an increased duration of restraint with rectal thermometry, which would provide at least a partial explanation for the increased stress reported between that and axillary thermometry. This information will not only provide insight on an uncontrolled factor in thermometry research, but it will also provide information that can be used to minimize the duration of clinical visits overall. 

## 2. Materials and Methods

Data was collected approximately weekly between November 2021 and May 2022 at a small animal hospital. Canine and feline pet owners with appointments for routine veterinary wellness care were approached and provided with information about the study. A confirmation of each animal’s wellness status and written owner consent to participate in the study were provided. Participation required acclimation to the clinic environment (22.14 °C ± 0.56 °C) for 15 min before initiating the study. Owners could elect to withdraw their pet at any point. Three canines and one feline were withdrawn prior to completing the full study at their owner’s request; this data was omitted. Participating pets (*n* = 186) included 114 canines and 72 felines. 

Animal demographic information is included in Table 1. Canine participants included 45 spayed females and 69 males, 12 of which were intact. All canine participants were at least 1 year old; the average age was 6.01 ± 3.87 years old (range 1.2–13.18 years). Canine breeds included twenty-five different pure and mixed breeds that varied from Chihuahua to Saint Bernard. Retrievers and retriever crosses were the largest breed group represented (33/114). The average canine weight was 22.57 kg ± 13.90 kg (range 3.58–54.88 kg). Animal body condition was scored on a 9-point scale. The average canine body condition score (BCS) was 6.02 ± 0.94 (range 4–8). Feline participant average age was 5.91 years ± 4.49 (range 1.01–15.69 years). Forty-nine out of 72 cats were classified as domestic short-haired breeds, 10 as domestic long-haired, 6 as domestic medium-haired, 5 as Siamese, and 2 as a Scottish Fold breed. Feline weight was 4.70 ± 1.25 kg (range 2.81–7.71 kg); BCS was 5.83 ± 1.15 (range 4–8). Animal body confirmation and chest size were classified as either small (<10 kg), barrel-chested, or keel-chested. Animal coat type was also classified as either smooth, short, medium, or long-haired for both canines and felines. 

**Figure 1:**
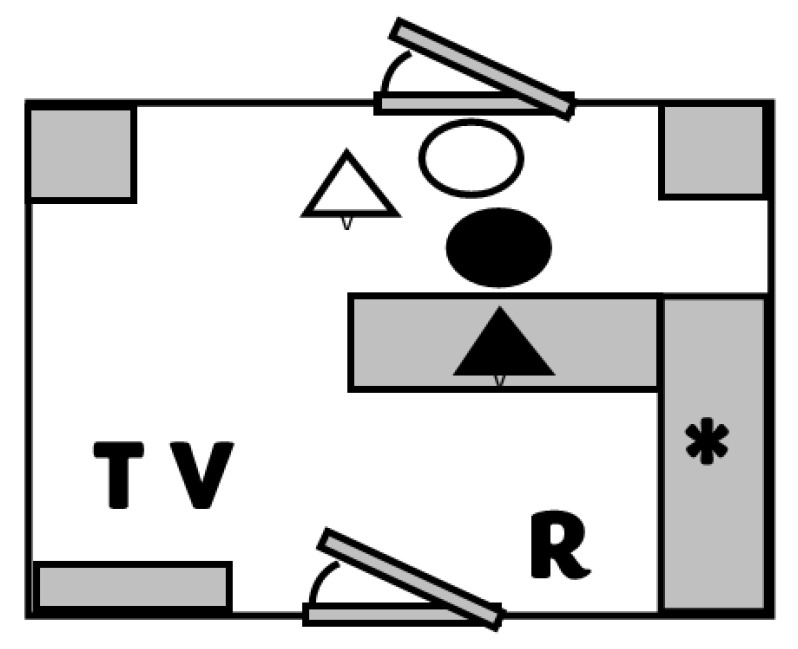
Schematic of the 10 × 14 foot study examination room. Letters represent the starting location of the veterinarian (V), technician (T), and recorder (R) for each trial. Pets are indicated as triangles, and owners are indicated as circles for the canine (white) and feline (black) patients. Grey boxes represent bench tops and furniture in the room. The star represents the location of the digital recording system where the temperature was recorded.

Using a crossover design, both rectal and axillary thermometry were performed successively on each pet. Each animal was randomly assigned to receive rectal or axillary thermometry first. Rectal thermometry was performed using a commercial digital thermometer (Vicks V912US; Proctor and Gamble, Cincinnati, OH, USA), and Axillary Thermometry was performed using a veterinary digital axillary thermometer (Mella Pro; Mella Pet Care, Chicago, IL, USA). Pets were brought into a 1400-square-foot examination room (22.14 °C ± 0.56 °C) individually by their owner, who remained in the room throughout the study. See Figure 1 for a schematic of the study design area. Canines remained on a lead on the floor, and felines were removed from carriers and lightly restrained on an examination table. The same veterinarian and licensed technician performed all the animal handling and thermometry. A third investigator remained near the door to monitor and record the timing. Markers on the floor indicated the veterinarian’s and technician’s starting locations. The veterinarian would verbally signal that timing should commence, and both the technician and veterinarian would approach the pet. The technician would restrain the animal in whatever fashion seemed most appropriate given the animal’s size and demeanor, and the veterinarian would perform the thermometry (rectal or axillary). As soon as the thermometer was positioned, the veterinarian would signal verbally to the timer, and the timing would stop. This was recorded as a Pre-temperature time (Pre-TempT). 

After the thermometry was completed, the veterinarian would provide another verbal cue indicating they were carried out. Recording would commence again, the thermometer would be removed from its position, the technician would return handling of the pet to the owner, and the veterinarian would travel to the digital record system and enter the temperature into the record. Once the record was entered, they would again verbally signal that recording should stop. This was recorded as a Post-temperature time (Post-TempT). The measurement time for each device varied; for that reason, the measurement time was not recorded. For analysis purposes, a standard 6-s recording time was used and added to the Pre-Temp and Post-Temp times to determine a presumptive total time (TTime). Once time values were recorded, all parties returned to their starting locations, and the process was immediately repeated. Pets who were initially randomly selected to receive rectal thermometry received axillary thermometry for the second round, and vice versa. 

Statistical analysis was performed as an aggregate for all pet participants and for canines and felines separately using a statistical software package (SigmaStat, San Jose, CA, USA). The normal distribution was confirmed using the Shapiro-Wilk normality test. Paired two-tailed *t*-tests were used to compare times (Pre-TempT, Post-TempT, and TTime) between rectal and axillary trials. A two-way repeated-measures analysis of variance (ANOVA) was performed where one variable remained the trial type (rectal or axillary) and the other tested the significance of trial order, breed type, BCS, sex, body confirmation, and hair type on each time value (Pre-TempT, Post-TempT, and TTime). The significance level was set at *p* values < 0.05. In the event of significance, a Bonferroni post-hoc multiple comparison test evaluated all pairwise interactions. All data reported are mean values ± standard deviation. 

## 3. Results

**Figure 2:**
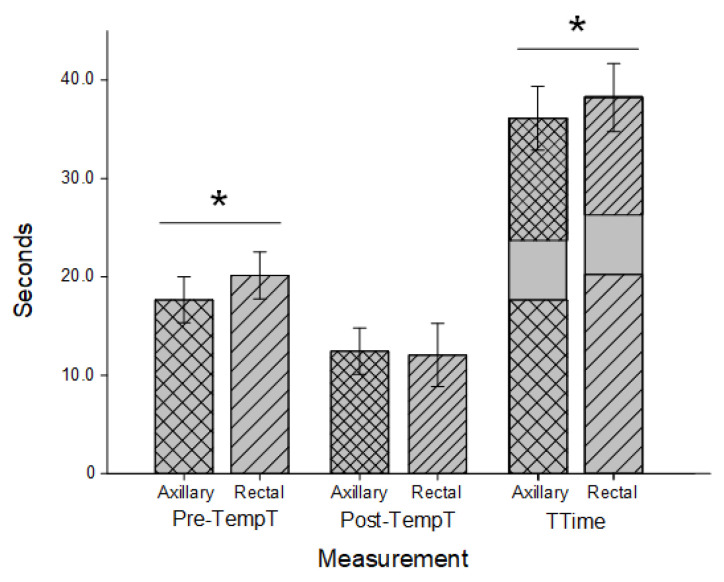
Timing for combined feline and canine thermometry trials. Axillary (cross hatched) and rectal (stripes) duration prior to temperature recording (Pre-TempT; wider spacing) and after a temperature recording until data entry was completed (Post-TempT; narrow spacing) in both canines and felines. Both times were added to a standardized 6-s recording time to determine the total time (TTime) for each trial. Both Pre-TempT and TTime were significantly shorter in the axillary trials than the rectal trials (*; *p* < 0.001).

**Figure 3:**
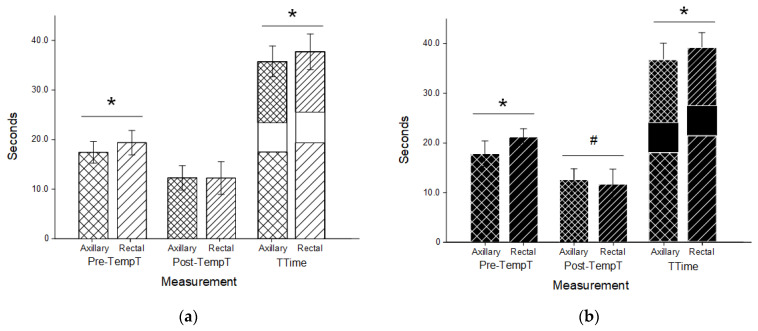
Timing for canine (**a**) and feline (**b**) thermometry trials. Axillary (cross hatched) and rectal (stripes) duration prior to temperature recording (Pre-TempT; wider spacing) and after a temperature recording until data entry was completed (Post-TempT; narrow spacing) in both canine (**a**; white) and feline (**b**; black) patients. Both times were added to a standardized 6-s recording time to determine the total time (TTime) for each trial. Both Pre-TempT and Ttime were significantly shorter in the axillary trials than the rectal trials in each species (*; *p* < 0.001). In felines, the Post-TempT in the axillary trials was longer than the rectal trials (#; *p* < 0.004).

Results for canines and felines combined are indicated in Figure 2 and are separated by species in Figure 3. For all pets, the Pre-TempT was shorter with the axillary trial (17.67 s ± 2.34 s) than the rectal trial (20.15 s ± 2.38 s; *p* < 0.001). For canines, the axillary Pre-TempT was 17.51 s ± 2.19 s and the rectal Pre-TempT was 19.43 s ± 2.45 s, and for felines, the axillary Pre-TempT was 17.92 s ± 2.57 s and the rectal Pre-TempT was 21.29 s ± 1.69 s (*p* < 0.001 for both). For all pets, the Post-TempT was not significantly different between the axillary and rectal trials (12.45 s ± 2.37 s and 12.06 s ± 3.20 s, respectively; *p* = 0.103). For canines, the Post-TempT for the axillary trial was 12.31 s ± 2.46 s, and the Post-TempT for the rectal trial was 12.27 s ± 3.31 s (*p* = 0.887). For felines, only the Post-TempT for the axillary trial was significantly longer at 12.67 s ± 2.22 s than the rectal Post-TempT (11.74 s ± 3.08 s; *p* = 0.004). With a standardized measurement duration of 6 sec, the TTime for all pets was shorter with the axillary trial (36.12 s ± 3.24 s) than the rectal trial (38.22 s ± 3.46 s; *p* < 0.001). For canines, the TTime for the axillary trials (35.82 s ± 3.13 s) was shorter than the rectal trial (37.70 s ± 3.61 s; *p* < 0.001). Similarly, for felines, the TTime was 36.59 s ± 3.39 s for the axillary trials and significantly longer at 39.03 s ± 3.08 s for the rectal trials (*p* < 0.001).

The order in which the trials took place did not significantly impact Pre-TempT, Post-TempT, or TTime for canines, felines, or all pets collectively (*p* > 0.05 for all). Canine participants were on the floor and felines on examination tables, making direct comparison difficult. Rectal Pre-TempT was shorter in canines than felines (*p* < 0.001), and consequently, the rectal TTime was also significantly shorter (*p* = 0.011). No other difference in timing was significant between canines and felines (*p* > 0.05 for rectal Post-TempT and all axillary times). Across all participants and within canines and felines individually, animal reproductive status (sex and intact vs. spayed) did not significantly affect Pre-TempT, Post-TempT, or TTime. Body condition score (BCS; 5.95 ± 1) did not differ significantly between canine (BCS 5.83 ± 1.15; range 4–8) and feline (BCS 6.02 ± 0.94; range 4–8) participants, nor did it significantly impact Pre-TempT, Post-TempT, or TTime for canines or felines of all pets collectively (*p* > 0.05 for all).

Canine breed variation and the small sample size of certain breeds made direct comparisons between breeds difficult. Body confirmation/chest classification as either small (<10 kg), barrel-chested, or keel-chested did not significantly impact Pre-TempT, Post-TempT, or TTime within or between rectal and axillary trials, nor did the designation of canine coat type as either smooth, short, medium, or long-haired (*p* > 0.05 for all). Similar results were determined for feline participants, with a few exceptions. We used post-hoc Bonferonni comparisons based on breed. All feline breed types demonstrated significant reductions in axillary Pre-TempT and TTime except the two felines designated as Scottish Fold breed (*p* = 0.51 and 0.76 for axillary vs. rectal Pre-TempT and TTime, respectively). Within the feline rectal trials, the TTime of domestic-long-haired breeds (41.91 s ± 0.93 s) was significantly longer than that of domestic-short-haired breeds (38.11 s ± 1.91 s; *p* = 0.019). No other difference was noted between breeds within or between the rectal and axillary trials. All feline participants were under 10 kg and, consequently, classified as small confirmation/chest size. Despite variation in breed designation, no difference was identified in or between felines with coats classified as either smooth, short, medium, or long haired (*p* > 0.05 for all).

## 4. Discussion

The total time (TTime) associated with axillary thermometry was shorter than that for rectal thermometry in both canine and feline participants. This is because in both species groups, the time leading up to the temperature measurement (Pre-TempT) was significantly shorter in the axillary temperature trials than the rectal temperature trials. For canine participants, the duration after the temperature was taken until it was recorded (Post-TempT) did not differ between axillary and rectal trials. In felines, the Post-TempT was actually shorter in the rectal trials, but not enough to compensate for the significant reduction in the axillary Pre-TempT. 

A number of factors could influence Pre-TempT. Pre-TempT was prior to thermometry, when the pet was approached and restrained for either thermometry method. In this study, efforts were made to control the space and distance traveled within species. Each clinic would likely have different results based on the size and arrangement of the facility. The personnel performing the measurements also remained constant. Presumably, in the average clinical environment, there would be some consistency in personnel and clinical spacing. Pet handling and restraint vary inherently based on breed, size, and animal demeanor. The crossover design was meant to minimize the effect of individual variation to the greatest extent possible. The experimental design was meant to provide insight on how the duration of handling for axillary thermometry differed from that of rectal thermometry. It is interesting that regardless of species, body confirmation, sex, coat type, and BCS, the axillary Pre-TempT was significantly shorter than the rectal Pre-TempT. Presumably, this is due to shorter restraint and handling times with axillary thermometry. Pre-TempT time for domestic-long-haired breeds was longer than for domestic-short-haired breeds; this may have been due to extra time being needed to identify the anus, which may be visually obscured in longer-haired animals. 

Feline participants were restrained on an examination table, and canine patients were on the floor. The location in the room and difference in restraint technique (extrinsic factors) may have contributed to the shorter Pre-TimeT and TTime for feline rectal thermometry than for canines. This difference may also be due to species variation. It is interesting that despite the variation in rectal thermometry between canines and felines, no difference was seen in axillary thermometry. In both species, however, axillary thermometry took significantly less time than rectal thermometry. 

In all cases, restraint served to maintain instrument position and stability. In rectal thermometry, restraint involved preventing cranial movement away from the thermometer, shifting the tail (as needed), and maintaining animal stability in the lateral and dorsal-ventral planes. Moving forward away from the instrument is a common action that can require additional force to prevent. Axillary thermometry involves abducting the forelimb enough to facilitate instrument placement, after which the pet is allowed to maintain a static position with stabilizing support. The time associated with maintaining additional movement restrictions and tail handling may have contributed to the longer rectal thermometry time.

Research on the effect of appointment duration is limited in veterinary medicine [20,21]. In dental literature, examination time is a strong predictor of appointment duration [22]. The clinical value of thermometry makes it a standard in most veterinary examinations and a likely contributor to appointment duration. Appointment duration not only impacts patient satisfaction, but it can also affect clinician time, the wait time for subsequent appointments, and the number of patients that can be seen in a given time [17,18,20,21]. In this study, animal restraint was performed by a licensed technologist and thermometry by a veterinarian. In many clinics, thermometry is performed by a single individual, with or without the support of an owner. The handling time would likely be increased for both axillary and rectal thermometry without the benefit of supportive restraint. 

Efforts to minimize stress in the veterinary clinic are an active area of research [14,15]. Patients who have had negative experiences are more fearful in subsequent veterinary appointments, and prolonged stress can adversely affect patient health [1,3,13,15]. Struggling and volatilization are likely to increase a patient’s sympathetic tone, affecting epinephrine, cortisol, and glucose levels, which may alter normal vital assessment parameters [16,19]. The extent to which variation in thermometry methods influences animal stress is unknown and outside the scope of this study. However, given the significant difference in duration between thermometry methods, this is an area of future research that should be considered. 

This study has a number of limitations. Conducting the study at one location as one prospective study, requiring continued owner approval, creates some selection bias. These results may not represent results at other locations or with other pets. The study took place on the same day of the week, based on investigator availability, and at relatively the same time. The crossover design of the study was meant to minimize individual variation. Although patients were randomly assigned the order of their measurements, the investigators were not able to be blinded. Comparing a random sampling of subgroups from either the rectal or axillary data produced similar results, and the level of significance in the data is compelling; however, we cannot eliminate the possibility of observer bias in this study. 

Device measurement time was standardized at 6 s but can vary between commercial devices. With technological changes, the advantage of this standardization was to focus on factors outside of the specific instrumentation used. This does not minimize the importance of device selection, which should be considered in the timing of thermometry. 

Duration and handling are not the only things to consider when selecting a thermometer. Accuracy is especially important [4,13]. Both thermometers used in this study meet federal accuracy standards in reporting the temperature at the sight of measurement. By convention, rectal thermometry is often used as an indication of “core temperature”, with clear parameters on the clinical significance of values at that location [4,5,13]. Studies have looked at the correlation between axillary thermometry and rectal temperatures in pets [23,24,25,26,27]. It may be that conversion factors are needed to interpret axillary temperatures or relate them to rectal or core temperatures. The effort to correlate alternate thermometry locations with rectal temperature may be replaced with studies determining the clinical significance of values at locations like the axilla. The purpose of this study was not to interpret the quality of the thermometer or the temperature values at a specific location, but rather to consider how the method of thermometry potentially affects handling and appointment duration. 

## 5. Conclusions

In a standardized clinical scenario, we found that the TTime associated with axillary thermometry was less than that of rectal thermometry in both canine and feline pets. This was due to the time associated with animal approach and restraint (Pre-TempT). This may have implications when considering clinic workflow, appointment duration, and patient handling time. 

## Figures and Tables

**Table 1:** Demographic information for canine and feline patient study participants.

Participants	Canines (*n* = 114)	Felines (*n* = 72)	Total (*n* = 186)
Sex	69 males (12 intact) 45 females	32 males 40 females (3 intact)	101 males (12 intact) 85 females (3 intact)
Age (years)	6.01 ± 3.87 (range 1.2–13.18)	5.91 ± 4.49 (range 1.01–15.69)	5.97 ± 4.09 (range 1.01–15.69)
Weight (kg)	22.57 ± 13.90 (range 3.58–54.88)	4.70 ± 1.25 (range 2.81–7.71)	15.65 ± 13.96 (range 2.81–54. 88)
Body Condition Score	6.02 ± 0.94 (range 4–8)	5.83 ± 1.15 (range 4–8)	5.95 ± 1 (range 4–8)
Body/Chest confirmation	75 Keel-Chested 21 Small (<10 kg) 18 Barrel-Chested	72 Small (<10 kg)	93 Small (<10 kg) 75 Keel-Chested 18 Barrel-Chested
Coat classification	34 Short 43 Medium 22 Long 15 Smooth	53 Short 6 Medium 13 Long 0 Smooth	87 Short 49 Medium 35 Long 15 Smooth

## Data Availability

Not applicable.
